# Should we increase the focus on diet when considering associations between lifestyle habits and deliberate self-harm?

**DOI:** 10.1186/s12888-020-02950-0

**Published:** 2020-11-25

**Authors:** Elizabeth Berg, Kay Wilhelm, Tonelle Handley

**Affiliations:** 1 Consultation Liaison Psychiatry Service, O’Brien Centre, St Vincent’s Hospital, 394 Victoria Street, Darlinghurst, NSW 2010 Australia; 2 Discipline of Psychiatry, Faculty of Medicine, University of Notre Dame, Sydney, NSW Australia; 3 School of Psychiatry, Faculty of Medicine, University of NSW, Sydney, Australia; 4 Centre for Rural and Remote Mental Health, University of Newcastle, Callaghan, Australia

**Keywords:** Suicidality, Deliberate self-harm, Depression, Lifestyle, Nutrition, Smoking

## Abstract

**Background:**

Despite increasing awareness of high rates of physical illness and poor lifestyle behaviours among patients with a history of repeated deliberate self-harm (DSH), there is little research on specific lifestyle factors that are potentially problematic for this group. This paper aims to explore the relationship between lifetime repeated DSH and certain lifestyle factors, including balanced meals, eating breakfast, consumption of ‘junk’ food, weight, exercise, substance/alcohol use, smoking and social support, in a cohort of patients who presented to the Emergency Department (ED) with suicidal ideation or DSH.

**Methods:**

From 2007 to 2016, data from lifestyle and mental health measures were collected from 448 attenders at an outpatient clinic for DSH or suicidal ideation following ED presentation. Lifestyle behaviours (Fantastic Lifestyle Checklist) and mental health (Depression and Anxiety Stress Scale), clinical diagnosis and number of previous DSH episodes were measured on arrival. The associations between lifestyle variables and the number of lifetime DSH episodes were examined.

**Results:**

Sex, age, depression symptoms, poor diet, and smoking were all associated with a higher average number of deliberate self-harm episodes across the lifespan. There were non-significant positive trends for the other poor lifestyle behaviours. There was no association between DSH episodes and diagnosis of depression or anxiety disorder. In a multiple linear regression model, the only factors that remained significant were age, smoking and eating balanced meals, however, the relationship between smoking and lifetime DSH was moderated by more immediate DSH behaviours.

**Conclusion:**

In this sample of patients referred to a service following presentation to the ED with acute mental health concerns, balanced meals and smoking were the lifestyle behaviours that were found to have the strongest independent association with repeated DSH across the lifespan.

## Background

While people presenting to Emergency Departments (EDs) with deliberate self-harm (DSH) are a heterogeneous group, studies have found these patients are more likely to have poorer long-term physical as well as mental health outcomes. Hawton et al. [1] longitudinal follow up study of 11,583 patients presenting to a hospital ED found that not only is DSH associated with increased risk of death by suicide, but also death from most natural causes, including respiratory disease, circulatory, neurological, endocrine, digestive, skin and musculoskeletal and connective tissue disorders. Another similar study [2] of 30,950 presenters with DSH reported deaths due to natural causes were 2–7.5 times more frequent than in the general population. Diseases of the circulatory and digestive systems were major contributors to high Years of Life Lost (YLL) from natural causes. They concluded that in the management of self-harm, clinicians should consider patients’ physical needs. The finding also suggests that there may be detrimental lifestyle factors associated with DSH that predispose these patients to premature death from all causes. Both studies suggested that lifestyle factors deserved greater attention in assessment and treatment of people presenting with DSH.

Previous research on lifestyle factors associated with DSH has focused on substance abuse and related disorders, including alcohol abuse and smoking. There is extensive literature reporting that substance (including alcohol and tobacco) abuse is strongly associated with DSH [3–8].

In addition to substance abuse, there is now also emerging interest in other lifestyle factors, including diet, exercise and obesity, and their role in mental health. Jacka and Berk [9] proposed that diet, exercise and smoking are independent risk factors for depression. A review [10] of lifestyle interventions related to suicide prevention noted that alcohol use, smoking and sedentary lifestyles were risk factors for all ages. They also noted that other risk factors varied by age group, with psychiatric symptoms, substance and alcohol use, weight and occupational difficulties being pertinent in adults and organic disease and poor social support being risk factors in the elderly. Similarly, a recent systematic review [11] revealed a complex relationship between obesity and suicidal behaviours, with some papers suggesting obesity is a protective factor against suicide fatality yet may increase the likelihood of non-fatal suicidal thoughts and attempts.

Regarding diet, the literature focuses on depression and suicidal behaviour, rather than DSH. Additionally, diet is a more difficult variable to define (and control) than exercise and hence the methodological variation between studies, with some comparing a diet high in ‘wholefoods’ with one high in ‘processed foods’ [12] and others exploring the effects of the Mediterranean diet [13]. Jacka and colleagues [14] conducted the world’s first randomised controlled trial comparing the effects of social support (known to benefit people with depression) and dietary interventions in the treatment of clinical depression. They demonstrated that one third of patients receiving support by a clinical dietician over 3 months met criteria for remission of major depression, compared to 8% of patients who received social support only. These results were directly proportionate to the extent of dietary change and not explained by weight loss or physical exercise alone. An observational study demonstrated similar results when exploring the dietary differences between suicide attempters versus non-attempters. The retrospective population-based study of almost 7000 adults [15], found that fruit, vegetables and meat, particularly fish/seafood, were significantly under-consumed in adults who were suicide attempters compared to non-attempters. This relationship remained significant after adjustment for various factors, including socioeconomic status, smoking, total caloric intake as well as medical and psychiatric illness. While most studies have focused on the nutritional components of an individual’s diet, there are a few studies that have examined specific dietary habits, such as eating breakfast. This is of interest given the evidence that eating breakfast significantly reduces one’s cardiovascular disease [16]. Eating breakfast is also correlated with decreased stress, depression and emotional distress [17, 18].

As lifestyle interventions are relatively new to psychiatry, there are no clear guidelines regarding formal assessment of problematic behaviours, such as poor diet. We have previously reported the Fantastic Lifestyle Checklist (FLC) [19] to be a valid tool for measurement of lifestyle behaviours in a sample of patients presenting to the Green Card Clinic (GCC) referred with suicidal behaviour or ideation, or non-suicidal self-injury [20]. We have also reported a significant difference between the overall FLC scores of people presenting with one and repeated DSH episodes to the GCC at St Vincent’s Hospital Sydney [21].

The purpose of our study is to further explore the relationship between the FLC diet and exercise items (nutrition, dietary habits exercise and weight) and lifetime history of DSH in this patient group. Our hypothesis was that poorer nutrition and dietary habits, infrequent exercise and obesity would be significantly associated with a higher number of lifetime episodes of deliberate self-harm. For the purposes of this study, deliberate self-harm encompasses both suicidal behaviours and non-suicidal self-injury.

## Method

### Procedure

This study used data collected from patients who attended the GCC at St Vincent’s Hospital. The details of the GCC are discussed more fully in previous papers [20–22]. In summary, following presentation to the ED with suicidal behaviour or ideation, or non-suicidal self-injury, all patients receive routine medical and psychiatric assessments (by an emergency doctor and psychiatry trainee). If deemed appropriate for discharge from hospital, the psychiatry trainee will consider referral to the GCC if: (1) the patient is not already under the care of their own psychiatrist/psychologist or community mental health team, (2) they speak English, and (3) they do not have a cognitive impairment, or major mental illness (schizophrenia, schizoaffective disorder, delusional or bipolar disorder). On arrival to their first clinic appointment, patients are asked by the clinic receptionist to complete several assessment measures, including those outlined below. Patients included in this study were also given information and the opportunity to consent to their de-identified data being used for the purposes of research. The St Vincent’s Human Research Ethics Committee approved the use of these data for this purpose.

### Participants

From 2007 to 2016, of the 665 patients attending their first GCC appointment, 448 provided complete data including current presentation, age, sex, marital status, past history of DSH, psychiatric diagnosis, and self-report measures (noted below). There were no significant demographic differences between those with complete data and those without. Over this time period, there were an additional 252 patients who either cancelled (*n* = 37), or did not attend (*n* = 215) their GCC appointment. There were no significant differences between people who attended their appointment (i.e. our sample in this paper) and those who did not. However, the minority of patients who cancelled their appointment were predominantly (27/37) female, and tended to be younger than those who did attend the GCC (mean age 27.0 ± 6.4 vs. 31.6 ± 10.9, F (2,927) = 4.38, *p* = .013).

The patients’ principal psychiatric diagnosis was recorded following clinical assessment by the GCC clinician (psychiatrist, psychiatry trainee, clinical psychologist or mental health clinical nurse consultant) and consensus by the team (following discussion of each case in weekly case review meetings). The clinical interviews were unstructured. Measures self-completed by patients (such as the Depression, Anxiety and Stress Scale; DASS) informed the clinicians’ view of the patient but were not used for diagnostic purposes. While many patients had comorbidities, the diagnosis recorded was the one that was the focus of treatment in GCC.

### Measures

The FLC is a 25-item measure that assesses 11 lifestyle domains using the acronym FANTASTIC (family, friends, activity, nutrition, toxins, alcohol, stress, sleep, personality type, insight and career). The current paper explored nine items within these domains, related to diet and health behaviours. These include receiving emotional support, exercise, eating balanced meals, eating breakfast, consuming excess sugar, salt, animal fats or junk food, weight, smoking, drug abuse, and alcohol. Items related to substance use, smoking, and social support were included as these factors may be potential confounds for the relationship between diet and DSH. Each item is scored on a 3-point Likert scale from 0 (*hardly ever*), 1 (*some of the time*), to 2 (*almost always*). There is some variation in wording depending on the item and some items are reverse scored. The checklist was provided to patients, who self-reported their lifestyle behaviours.

The DASS21 Item Version [23] measures three negative emotional states (depression, anxiety and stress). The two scales assessing depression and anxiety (7 items each) were used in this study. The depression scale assesses dysphoria, hopelessness, devaluation of life, self-deprecation, lack of interest, anhedonia and inertia. The anxiety scale assesses autonomic arousal, muscle tension, situational anxiety and subjective experience of anxiety. Respondents rate the extent to which they have experienced each state over the past week on a 4-point Likert scale ranging from 0 (*never*), 1 (*sometimes*), 2 (*often*) to 3 (*almost always*). Total scores for each subscale range from 0 to 21, with a score above 11 on the depression subscale or 8 on the anxiety subscale being considered “severe.” This self-report scale was completed by patients independent of the diagnosis they received from the clinician who assessed them.

### Outcome measure

The outcome measure for this study was the total number of self-reported lifetime DSH episodes, including the current presentation. Suicidal intent was not measured, therefore DSH episodes in this study include both suicide attempts and non-suicidal self-injury.

### Statistical analyses

The data were analysed using the Statistical Package for the Social Sciences (SPSS, v22, IBM Corporation, 2013). Descriptive statistics were used to quantify baseline outcome measures and other variables (including demographics, psychiatric diagnosis and lifestyle factors). For the purpose of analysis, several dichotomous variables were created. The two groups in the marital status variable included single/separated/divorced/widowed and married/defacto. The diagnosis variable was collapsed into four categories: depressive disorder, anxiety disorder, substance use disorder, and all other diagnoses. In forming dichotomous variables from the lifestyle factors, the decision was made to isolate the most extreme negative response. For example, the balanced meals variable was divided into the two groups: (1) ‘*hardly ever’* and (2) ‘*some of the time’* or ‘*almost always’*. The exceptions to this rule were the smoking and drug abuse variables. The smoking variable was divided into the two groups: (1) smokers (‘*occasional use’* or *‘daily use’*), and (2) non-smokers. Likewise, the drug abuse variable was divided into two groups of people who abuse drugs: (1) *‘some of the time’* or *‘frequently’* and (2) *‘never or seldom’*.

The rationale for this was that the middle response options for the smoking and drug abuse variables were particularly difficult to quantify, and considering there is no amount of smoking or drug abuse that is considered ‘safe’ by national guidelines (unlike alcohol), this seemed to be the most logical way to divide these variables into two groups. Additionally, research shows that risk of death from suicide tends to be primarily associated with amount of alcohol consumed per drinking day, rather than drinking frequency or overall alcohol consumption, which supports guidelines that limit consumption to 2 drinks or less per drinking day [24].

Basic descriptive statistics were used to describe participant characteristics (counts and percentages for categorical variables, means and standard deviations for continuous variables). One-way ANOVA and correlation analyses were used to explore the relationship between the number of lifetime DSH episodes and a range of variables including sex, marital status, depression, anxiety, substance use disorder (SUD) and the list of dichotomous lifestyle variables (as described above). Where demographic and diagnostic variables were found to have a significant univariate relationship with the number of lifetime DSH episodes (*p* < *.05*) in the ANOVA or correlation analyses, they were included in a multiple linear regression model to assess the predictive ability of the various lifestyle and demographic variables. This was run as a hierarchical model with two steps; the first step included only those demographic and diagnostic variables that showed a significant univariate relationship with the outcome. The second step included all of these variables, as well as a variable representing the reason for the patient’s presentation to the ED. This was due to a significant relationship between the patient’s reason for presentation and the outcome, which is described further in the results section.

## Results

Of the total sample, 107 (23.9%) had never engaged in DSH, 162 (36.2%) reported one previous episode of DSH and 179 (40.0%) reported 2 or more previous episodes of DSH. The reasons for their ED presentation were overdose (40.8%), suicidal ideation (41.7%), cutting (10.3%), hanging (1.6%), jumping (2.0%) carbon monoxide poisoning (0.2%) and other (3.3%). The majority of patients (58.1%) presented to the ED with DSH, with a smaller proportion (41.9%) presenting with SI. Characteristics of the sample are described in Table 1. Demographic, clinical and lifestyle data were explored by the number of lifetime DSH episodes. There was a significant sex difference, with females having a higher average number of DSH episodes than males (2.0 ± 2.8 vs. 1.4 ± 1.9, F (1,447 = 6.7, *p* = .01). Similarly, there was a significant effect for age, with younger age being associated with a higher number of DSH episodes (*r* = − 0.17, *p* < .01).

**Table 1:** Demographic characteristics, and number of lifetime deliberate self-harm episodes by sample characteristics (*n* = 448)

Characteristic	% (n)	Mean DSH episodes (SD)^a^
Sex		
Male	42.9 (192)	1.4 (1.9)
Female	57.1 (256)	2.0 (2.8)**
Age	31.7 (10.6) ^b^	−0.17**^c^
Marital status		
Married/de facto	15.9 (71)	1.3 (1.5)
Single	84.2 (377)	1.8 (2.6)
Depressive Disorder		
Yes	32.8 (146)	1.8 (3.2)
No	67.4 (302)	1.7 (2.0)
Anxiety Disorder		
Yes	10.8 (48)	1.3 (1.2)
No	89.3 (400)	1.8 (2.6)
Substance Use Disorder		
Yes	19.2 (86)	2.0 (1.9)
No	80.8 (362)	1.7 (2.6)
DASS depression	13.2 (5.6)^b^	0.11*
DASS anxiety	9.5 (5.6)^b^	0.05
Alcohol		
≤2 drinks/day	75.9 (340)	1.8 (2.8)
> 2 drinks/day	24.1 (108)	1.6 (1.4)
Abuse of prescribed or unprescribed drugs		
Seldom/never	55.8 (250)	1.5 (2.1)
Sometimes/frequently	44.2 (198)	1.9 (2.8)
Smoker		
Never	41.3 (185)	1.3 (2.6)
Daily or occasional use	58.7 (263)	2.0 (2.5)**
Receive emotional support		
Sometimes/often	74.6 (334)	1.6 (2.2)
Hardly ever	25.4 (114)	2.1 (3.1)
Weight		
Within 8 kg of ideal weight	79.5 (356)	1.7 (2.7)
Not within 8 kg of ideal weight	20.5 (92)	1.7 (1.8)
Consume excess sugar, salt, animal fats or junk food		
Never/sometimes	84.4 (378)	1.7 (2.4)
Almost always	15.6 (70)	1.7 (2.6)
Eat breakfast		
Sometimes/often	66.7 (299)	1.6 (2.3)
Hardly ever	33.3 (149)	1.9 (2.8)
Eat balanced meals		
Sometimes/often	79.5 (356)	1.6 (1.9)
Hardly ever	20.5 (92)	2.3 (4.0)**
Active exercise for 30mins		
Sometimes/often	66.7 (299)	1.6 (2.6)
Hardly ever	33.3 (149)	1.9 (2.2)

^a^statistical significance was established by a one-way ANOVA, unless otherwise indicated. ^b^values presented are mean and standard deviation. ^c^values presented are pearson’s correlation coefficients (*r*). *p < .05, **p < .01. *DASS* Depression, Anxiety and Stress Scale. Categorical variables were analysed one-way ANOVA; continuous variables were analysed by correlation

There was no significant association between the number of DSH episodes and marital status or diagnosis of depression, anxiety or substance use disorder. However, greater frequency of DSH episodes was associated with higher DASS depression scores, with a weak effect size (see Table 1). The tobacco smoking and diet were only lifestyle variables that demonstrated a positive relationship with the number of DSH episodes. Current tobacco smokers and those who reported they “hardly ever” ate balanced meals both reported more lifetime DSH episodes.

All the other lifestyle variables, including excessive alcohol, minimal emotional support unhealthy body weight, high junk food consumption, not exercising and not eating breakfast demonstrated non-significant effects.

Although the focus of this analysis was to explore the relationship between lifetime DSH and current lifestyle behaviours, an analysis was conducted to determine whether lifestyle behaviours were associated with the reason for the current presentation (DSH or suicidal ideation). Only two lifestyle behaviours were significantly associated; those who presented with DSH were more likely to be current tobacco smokers (64.7% vs. 52.5%, χ^2^_(1)_  = 6.58, *p*  =.010), and to use illicit drugs (50.8% vs. 38.8%, χ^2^_(1)_ = 6.11, *p*  =.013). Those who presented with suicidal ideation in their current episode also had higher DASS depression (14.6+/− 5.0 vs. 12.1 +/− 5.8, *F* = 22.1, *p <* .001), and anxiety (10.5+/− 5.3 vs. 8.8+/− 5.7, *F* = 9.9, *p* = .002) than those who presented with DSH.

As substance use is likely to affect other lifestyle factors, we compared those with and without a diagnosis of substance use disorder. There was evidence of co-occurrence between several key behaviours; of the 86 participants with a diagnosed substance use disorder, 65 (75.6%) also currently smoked tobacco. Similarly, of the 90 people who “hardly ever” ate balanced meals, 63 (70.0%) were current smokers.

Table 2 presents the multiple linear regression model (F = 5.446, *p*  <.001, *R*^2^ = .062) that includes age, sex, DASS depression, tobacco smoking, and balanced meals. Smoking, eating balanced meals and age were the only variables that were significantly associated with higher lifetime DSH episodes. Due to the association between reason for presentation (DSH or suicidal ideation) and tobacco smoking and DASS scores, the regression was re-run with this variable included (F = 7.688, *p* < .001, *R*^2^ = .101). While age and eating balanced meals remained significant in this model, DASS depression became significant, and the effect of tobacco smoking was no longer significant. This suggests that current smoking status and DASS depression are influenced by current presentation and emotional state.

**Table 2:** Multiple linear regression model with age, sex, depression and lifestyle variables with lifetime deliberate self-harm episodes (*n* = 448)

	Unstandardised beta	Standardised beta	***p***
**Step 1**			
Age	−0.03	−.012	.013
Sex (female)	0.41	0.08	.106
DASS depression	0.02	−0.05	.314
Smoker (occasional or daily use)	−0.50	−0.10	.047
Hardly ever eat balanced meals	−0.62	−0.10	.044
**Step 2**			
Age	−0.03	−0.12	.013
Sex (female)	0.34	0.07	.180
DASS depression	0.05	0.10	.042
Smoker (occasional or daily use)	−0.34	−0.07	.155
Hardly ever eat balanced meals	−0.63	−0.10	.037
Reason for presentation (deliberate self-harm)	1.05	0.21	<.001

Note: step 1 of the regression contains age, sex, DASS depression, smoking status, and balanced meals; step 2 of the regression contains age, sex, DASS depression, smoking status, balanced meals, and the reason for presentation to the ED. *DASS* Depression, Anxiety and Stress Scale

## Discussion

The aim of this study was to add to existing data on lifestyle behaviours of patients presenting to ED with SI or DSH, using data from a further cohort of GCC attenders. We focused on diet as an area of growing interest that has not been investigated in DSH groups and because the FLC has a number of specific diet and diet-related items along with a range of variables, including demographics, psychiatric diagnoses, and DASS scores gathered at their first visit.

Results showed a significant relationship to sex and age, with younger people and females reporting more lifetime episodes of DSH. Higher DASS depression scores were associated with more frequent DSH episodes, rather than clinical diagnosis of depressive or anxiety disorders per se. This is similar to the findings from the previous cohort [22] where DASS depression scores were higher in those with repeated DSH episodes than those presenting with a single DSH attempt. While clinical diagnosis of SUD was not associated with higher rates of DSH, it was strongly related to other health behaviours including smoking, which in turn was related to the outcome, suggesting an indirect relationship between substance misuse and DSH.

In terms of lifestyle factors explored, there was a significant relationship between the diet and smoking variables and repeated DSH and a non-significant trend on all other lifestyle variables, including emotional support, breakfast, drug use, junk food, weight and alcohol. However, using a multiple linear regression model, we demonstrated that balanced meals, current smoking and younger age were the only variables found to be significantly associated with lifetime episodes of DSH and such variables as eating junk food and obesity were not. This is an interesting finding as ‘eating balanced meals’ and smoking were more significant items for this patient group than items such as substance and alcohol use, which are more likely to be assessed. We know from our findings that there is considerable overlap between those who were smokers and those who did not have balanced meals [25] and speculate that these two questions may be a proxy for poor lifestyle behaviours in general, in keeping with the previously reported ‘poor health behaviours’ component in a factor analysis of the Fantastic Lifestyle Checklist [20].

While current smoking was significantly related to the number of lifetime DSH episodes, this effect was no longer significant when the reason for the current presentation to the ED was adjusted for. This suggests that smoking may be more strongly related to current emotional state, rather than lifelong DSH behaviours, although clinically, we had the impression that our attenders were long term smokers and speculate that some restarted smoking in response to stress rather than taking up smoking for the first time. However, we did not explore this. Conversely, DASS depression was not related to number of DSH episodes in the first step of the regression, but became significant when reason for ED presentation was included. Our findings imply that once current emotional distress is accounted for, there is a chronic aspect of depressive symptoms that is more strongly related to lifelong DSH.

The importance of diet and nutrition in the assessment and management of patients with DSH requires further attention. Given the emerging evidence of the importance of certain elements of a balanced diet, including fruit, vegetables and fish, these dietary elements need to be explored in relation to history of repeated DSH. Future research utilising a more detailed dietary assessment may be beneficial. Lifestyle interventions hold promise because of their ease of implementation and cost-effectiveness, as well as their known association with depression. They also have the potential to prevent the excessive morbidity and mortality resulting from high rates of physical illness among patients with a history of suicidal behaviour. Jacka has used the term ‘nutritional psychiatry’ to highlight the burgeoning clinical and research interest [14].

Having previously argued for importance of asking about smoking [25] in assessing people presenting with suicidality, we now suggest that asking about ‘balanced meals’ can segue into discussing nutrition in general (the lifestyle checklist also asks about consumption of junk food and breakfast-eating habits). The two questions may provide a simple ‘window of opportunity’ to discuss lifestyle issues with people presenting with DSH before they develop further mental and physical health problems.

The review of lifestyle interventions related to suicide prevention [10] concluded that “lifestyle behaviours including cigarette smoking, alcohol use, and sedentary lifestyle are associated with suicide risk in all age groups” and for adults, “psychiatric symptoms, substance and alcohol abuse, weight, and occupational difficulties seem to have a significant role in suicide risk. However, most of the research cited in adults focuses on people with a history of chronic mental illness. Our group of attenders were almost exclusively in the adult group but did not have chronic mental illness (such as schizophrenia or bipolar disorder) with concomitant symptoms of poor diet, metabolic syndrome and other chronic health issues. Furthermore, less than half had a primary clinical diagnosis of depression, anxiety or adjustment disorders: the others were more representative of people arriving at an ED in crisis, following including acute stress or substance misuse.

While there is a growing body of research into lifestyle risk factors associated with increased suicide risk, there is relatively little on the differences in lifestyle behaviours of people who repeatedly self-harm compared to non-repeaters. This is important given the reports of increased mortality and morbidity from a range of natural causes [1, 2] in people presenting to EDs with DSH and the links between repeated DHS and poorer lifestyle behaviours documented in our earlier study [21]. In that study of an earlier cohort from the same clinic in 1998 to 2005, we have reported that nearly half the group (42.7%) reported repeated DSH episodes and had the poorer scores on the FANTASTIC lifestyle checklist than either single DSH episode or SI groups. We suggested that repeat self-harmers, may do better an approach aimed at ‘lifestyle change’ rather than based on current psychological stressors. In this later cohort, we sought to pursue the issue of lifestyle further, with particular focus on diet as this is an area of growing interest that has not been investigated in DSH groups.

A limitation of this study design is that data were collected when the patients were distressed and/or depressed and not repeated later (these patients are relatively mobile and difficult to follow up). Additionally, this study included only people who presented to hospital and the GCC, consented to provide their data, and completed all measures, thus excluding those who did not. This gives a potential bias but interestingly, the nonattenders were more likely to be slightly younger females. In addition, we measured only the patients’ primary psychiatric diagnosis, and did not assess any secondary diagnoses or comorbidities. Finally, we measured lifetime DSH, but did not assess level of suicidal intent in previous episodes, so are not able to differentiate between suicide attempts and non-suicidal self-injury. However, this can be difficult to determine retrospectively and we consider that our approach reflects clinical reality. We believe the issues raised reflect those seen in services for people presenting to ED with DSH and are of clinical interest from an individual and public health perspective. This paper also has considerable strengths. To our knowledge, it is the first to quantify the specific lifestyle behaviours associated with the number of lifetime DSH episodes. We were able to successfully identify poor diet and cigarette smoking as the strongest lifestyle correlates of DSH, which may inform holistic approaches to reduce or prevent DSH behaviours among those experiencing psychological crises.

## Conclusions

These findings can contribute to a first step in evaluating the impact of interventions relating to such lifestyle interventions as improving diet, exercise, social inclusion, and ceasing smoking and other substances for individuals presenting with suicidality. It also provides an opportunity for more education to patients about the importance of diet, which seems particularly important given the high rates of metabolic and cardiovascular disease among patients with a history of DSH [1, 2]. There is enormous potential to be gained in understanding the impact of lifestyle behaviours on mental health and educating and empowering people to make lifestyle changes, not only to prevent them developing non-communicable disease, such as diabetes, but also for their emotional wellbeing and sense of agency.

## Data Availability

The datasets used and/or analysed during the current study are available from the corresponding author on reasonable request.
